# Editorial: “Metal Isotope Analytical Chemistry for Geological and Environmental Sample”

**DOI:** 10.3389/fchem.2021.689873

**Published:** 2021-05-11

**Authors:** Chaofeng Li, Zhaochu Hu, Lu Yang, Gangjian Wei

**Affiliations:** ^1^ Institute of Geology and Geophysics, Chinese Academy of Sciences (CAS), Beijing, China; ^2^ State Key Laboratory of Geological Processes and Mineral Resources, China University of Geosciences, Wuhan, China; ^3^ Institute for National Measurement Standards, National Resarch Council Canada, Ottawa, Ont, Canada; ^4^ Guangzhou Institute of Geochemistry, Chinese Academy of Sciences, Guangzhou, China

**Keywords:** metal isotope analysis, TIMS, MC-ICP-MS, LA-ICP-MS, SIMS

**Editorial on the Research Topic**

**Metal Isotope Analytical Chemistry for Geological and Environmental Sample**

Metal isotopes are important tracers for various geological and environmental processes. Accurate determination of metal isotope ratios is a hot topic at present, evidently from the rapid growth in the number of related publications in geoscience and environmental sciences. High precision isotopic analysis techniques based on modern inorganic mass spectrometry (TIMS, MC-ICP-MS, SIMS, and LA-ICP-MS/MC-ICP-MS) have been developed, greatly driving the developments in geoscience and environmental science over the past half century. Among these activities, *in situ* isotopic analysis and dating analytical techniques for special minerals (e.g., zircon, garnet, clinopyroxene, pyrite, chalcopyrite and ferromanganese nodule) based on LA-MC-ICP-MS, LA-ICP-MS and SIMS are rising fast in geoscience due to their high spatial resolution, fast analysis, small sample size, minimal sample preparation and low contamination. To obtain high precision, many key technical issues must be thoroughly investigated such as matrix effects, sources of interfering substances, isotopic fractionation correction, matrix-match standards and sensitivity improvements. In addition, sample purification techniques with low blank, low cost, high recovery, high selectivity, and high sample throughput are crucial for high precision metal isotope ratio measurements for bulk analysis in geological samples.

The Cu isotope ratio in Cu-dominated minerals is a powerful tool to trace the ore-forming source and study metallogenic system evolution. In previous studies, rigorous sample purification is indispensable, but it is time-consuming and tedious. One paper in this special issue has reported a novel analytical method for the direct determination of Cu isotope ratios without column chemistry. The influence of matrix elements on the final Cu isotope ratios can be corrected by using a C-SSBIN with Ga as an internal standard. The proposed method has a significant advantage for the economical and efficient determination of Cu isotopic ratios in Cu-dominated minerals.

Due to large relative mass difference between ^6^Li and ^7^Li, Li isotope ratio is of great interest in many fields including geochemistry, astrophysics and the nuclear industry. A new sample preparation technique for Li isotope ratio measurements based on a single-column AGMP50 cation resin is reported in one of collections in this special issue. The proposed method is rapid and ideally suited for Li separation from complex geological sample matrix prior to MC-ICP-MS analysis. The proposed method shows great potential in study conventional silicate materials and trace both high-temperature magma processes and low-temperature weathering processes.

Reference materials (RMs) play an important role in isotopic analysis for the validation of analytical methods employed to ensure the quality of data. The commonly used Cu isotopic standard NIST SRM976 (National Institute of Standards and Technology) is no longer available. One paper in this special issue reported characterization of four new commercially available Cu metal materials (SSC-1, SSC-3, SSC-4 and CUPD-1), which were proposed as reference materials for high precision Cu isotopic analysis by LA-MC-ICP-MS. There is also an increasing demand for natural clinopyroxene reference materials for Sr isotopic microanalysis. Based on two year *in situ* Sr isotopic analyses by LA-MC-ICP-MS, six potential clinopyroxene materials with moderate Sr content ranging from 100 to 350 μg g^−1^ from South Africa and China were identified as excellent potential RMs for Sr isotopic microanalyses in one of the papers. These potential reference materials are sufficient to distribute to the scientific community via contacting the corresponding author.

Zircon U-Pb dating has played the most important role in Earth and solar system science in constraining the ages of a wide variety of rocks. SIMS is one of the most important analytical tools for zircon U-Pb dating. In this special issue, a study about the influence of relative analytical position in zircon on U-Pb age dating by SIMS was performed. U-Pb age deviations as high as around 10% were found on the left and right side with overlap in the raster area. This study calls for re-examination for the previous SIMS U-Pb dating results on core-rim dating strategy, and provides a calibration protocol to correct the relative position effect.

In addition, in this special issue a review paper on analytical methods for Os isotope ratio measurements and Re-PGE mass fractions in geological samples is presented. This review is a comprehensive reference for researchers to find the state-of-the-art developments in measurement methods for ^187^Os/^188^Os ratios and Re-PGE mass fractions in geological materials.

Five full papers and one review are published in this special issue, and we regret that many other excellent manuscripts were not included due to the limitation of the number of pages available for this special issue. We thank all authors who have contributed to this special issue and appreciate all reviewers’ efforts to maintain the high quality of reviewing process for these papers. Special thanks are due to Dr. Aliki Moysiadi and the Editorial Board of Frontiers in Chemistry for giving us this opportunity to organize this issue. It is hoped that this special issue will help accelerate future developments in metal isotopic analysis for geological and environmental samples. We hope that you would enjoy reading this collection of articles.

